# Interfacial Properties, Wettability Alteration and Emulsification Properties of an Organic Alkali–Surface Active Ionic Liquid System: Implications for Enhanced Oil Recovery

**DOI:** 10.3390/molecules27072265

**Published:** 2022-03-31

**Authors:** Bennet Nii Tackie-Otoo, Mohammed Abdalla Ayoub Mohammed, Hazman Akmal Bin Mohd Zalghani, Anas M. Hassan, Pearl Isabellah Murungi, Grace Amabel Tabaaza

**Affiliations:** 1 Petroleum Engineering Department, Universiti Teknologi PETRONAS, Seri Iskandar 32610, Malaysia; hazman.akmal_24316@utp.edu.my (H.A.B.M.Z.); pearl_20001549@utp.edu.my (P.I.M.); 2 Centre of Research in Enhanced Oil Recovery, Universiti Teknologi PETRONAS, Seri Iskandar 32610, Malaysia; 3 Petroleum Engineering Department, Khalifa University of Science, Technology and Research, Abu Dhabi P.O. Box 127788, United Arab Emirates; anas.hassan@ku.ac.ae; 4 Chemical Engineering Department, Universiti Teknologi PETRONAS, Seri Iskandar 32610, Malaysia; grace_20000207@utp.edu.my

**Keywords:** surface-active ionic liquid, organic alkali, interfacial tension, wettability alteration, emulsification, alkali–surfactant flooding

## Abstract

Combinatory flooding techniques evolved over the years to mitigate various limitations associated with unitary flooding techniques and to enhance their performance as well. This study investigates the potential of a combination of 1-hexadecyl-3-methyl imidazolium bromide (C_16_mimBr) and monoethanolamine (ETA) as an alkali–surfactant (AS) formulation for enhanced oil recovery. The study is conducted comparative to a conventional combination of cetyltrimethylammonium bromide (CTAB) and sodium metaborate (NaBO_2_). The study confirmed that C_16_mimBr and CTAB have similar aggregation behaviors and surface activities. The ETA–C_16_mimBr system proved to be compatible with brine containing an appreciable concentration of divalent cations. Studies on interfacial properties showed that the ETA–C_16_mimBr system exhibited an improved IFT reduction capability better than the NaBO_2_–CTAB system, attaining an ultra-low IFT of 7.6 × 10^−3^ mN/m. The IFT reduction performance of the ETA–C_16_mimBr system was improved in the presence of salt, attaining an ultra-low IFT of 2.3 × 10^−3^ mN/m. The system also maintained an ultra-low IFT even in high salinity conditions of 15 wt% NaCl concentration. Synergism was evident for the ETA–C_16_mimBr system also in altering the carbonate rock surface, while the wetting power of CTAB was not improved by the addition of NaBO_2_. Both the ETA–C_16_mimBr and NaBO_2_–CTAB systems proved to form stable emulsions even at elevated temperatures. This study, therefore, reveals that a combination of surface-active ionic liquid and organic alkali has excellent potential in enhancing the oil recovery in carbonate reservoirs at high salinity, high-temperature conditions in carbonate formations.

## 1. Introduction

Chemical-Enhanced Oil Recovery (cEOR) methods have proven to be very efficient in mobilizing and extracting residual and remaining oils from matured reservoirs [1]. Primary and secondary oil recovery techniques leave a momentous volume of oil unrecovered, owing to trapping by capillary forces and unstable displacement fronts. The chemical flooding methods, such as the injection of alkali, surfactant, polymer, foam and low-salinity water, are used to recover trapped oil. Various hybrid techniques have been developed over the years to enhance the performances of individual techniques, as well as mitigate their limitations [2]. Alkali-augmented surfactant (AS), alkali-augmented polymer (AP), polymer-augmented surfactant (SP) and alkali–surfactant–polymer (ASP) flooding are some of the hybrid techniques deployed for recovery [3]. In addition, nanoparticles are deployed to augment various flooding methods, like surfactant nanofluids, nanoparticle–nanoparticle–surfactant foam, polymeric nanofluids and smart nano-waterflooding [2,4,5,6,7,8].

Despite the promising nature of various chemical floodings and their hybrid techniques, the chemicals deployed have associated limitations that inhibit their worldwide application [9]. The inorganic alkalis conventionally deployed, like NaOH and Na_2_CO_3_, cause severe scaling problems, which impair reservoir permeability and lead to loss of production capacity [10,11,12,13]. Conventional surfactants have serious environmental concerns due to their low biodegradability and biocompatibility. Some of these surfactants precipitate in the presence of divalent cations and lose their functionality [14]. Several scholars have investigated and suggested alternatives in the literature [9]. They have proposed a switch to using organic alkalis as alternatives to inorganic alkalis. Monoethanolamine (ETA) has proven to be one of the most promising organic alkalis that have undergone extensive studies [10,11,12,13,15,16,17]. Renewable resource-based surfactants have also been proposed as alternatives to petrochemical-based surfactants due to their high biodegradability and biocompatibility [18,19,20,21,22,23,24,25,26,27]. Surface-active ionic liquids (SAILs) have also been proposed for surfactant application in harsh reservoir conditions (high-temperature and high-salinity reservoirs) [28,29].

Despite the economic significance of carbonate reservoirs (i.e., containing about 60–65% of the world’s remaining oil-proven reserves [30,31,32]), their oil recovery poses a great challenge. Anionic surfactants are widely used in sandstone reservoirs due to their negative headgroup and, hence, adsorb less on the sand surface (surface charge of sand being negative). Cationic surfactants, on the other hand, have positive headgroups and are more suitable for residual oil recovery in carbonate rocks. Cetyltrimethylammonium bromide (CTAB), a cationic surfactant, has been proven by recent investigations to exhibit better wettability alteration capability on carbonate rock surfaces than anionic surfactants [33]. Furthermore, the widely deployed Na_2_CO_3_ in cEOR has limited applications in carbonate due to severe scaling problems in the presence of gypsum and anhydrite [34]. Sodium metaborate (NaBO_2_) has been deployed as one of the alternative inorganic alkalis. It elevates pH without substantial permeability impairment. It also has high resistance to divalent cations and is a better alternative for carbonate reservoir application [35,36]. A combination of NaBO_2_ and CTAB has been proven to exhibit synergistic performance in IFT reduction and wettability alteration, as well as the formation of stable emulsions.

**Figure 1:**
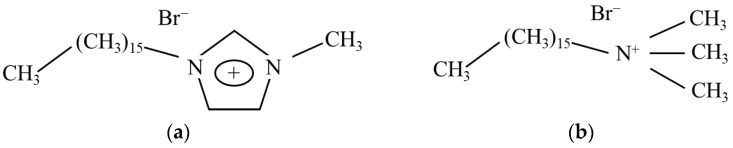
Chemical structures of (**a**) C_16_mimBr and (**b**) CTAB.

Among the SAILs that have been investigated as alternatives to conventional surfactants, 1-hexadecyl-3-methylimidazolium bromide (C_16_mimBr) has been studied comparatively to CTAB by Nandwani et al. [37]. C_16_mimBr is considered a cationic surfactant, and this comparative study is justified by the similarity in its structure and aggregation behavior to that of CTAB. The structures of C_16_mimBr and CTAB are shown in Figure 1. The two surfactants have the same hydrophobic chain lengths and counterions. C_16_mimBr exhibited superior interfacial properties to CTAB in high-salinity conditions [37].

Therefore, it has been proven in the literature that ETA and C_16_mimBr have excellent potential as alternatives to inorganic alkalis and cationic surfactants in carbonate EOR applications. Nevertheless, a combination of these two alternative chemical agents that will yield better oil recovery through a synergistic performance has not be reported in the literature yet. Herein, an AS formulation comprising ETA and C_16_mimBr is proposed. This study focused on investigating the synergies that exist between ETA and C_16_mimBr in enhancing oil recovery. First, the aggregation behavior of C_16_mimBr is revisited and studied in comparison to CTAB. The proposed AS formulation’s IFT reduction and wettability alteration capabilities are studied in comparison to a conventional AS formulation composed of NaBO_2_ and CTAB. Finally, the interfacial properties of the formulation are confirmed through emulsification studies.

## 2. Materials and Methods

### 2.1. Materials

The details of the various materials used in this study are summarized in Table 1. The study utilized ETA and NaBO_2_ as alkalis and C_16_mimBr and CTAB as surfactants. Synthetic brine was prepared using nine salts. The brine composition and properties are presented in Table 2. A light crude oil from a Malaysian oil field was deployed as the oleic phase. Its composition and properties are also summarized in Table 2. The chemicals were used as received, and the deionized water was not purified further. The preparation and dilution of various chemical solutions and brine were done with deionized water.

An outcrop from a Malaysian carbonate formation was utilized for the wettability alteration studies. Thin slices of the rock sample with dimensions 20 × 20 × 3 mm were made and trimmed for contact angle measurements. The crushed and ground parts of the carbonate sample were then characterized using X-ray fluorescence (XRF) (model Bruker; S8 Tiger) and X-ray diffraction (XRD) (model X’Pert^3^ Powder & Empyrean, PANanalytical). Fourier-transform infrared (FTIR) spectroscopy was then conducted with an FTIR spectrophotometer (Perkin Elmer Spectrum 2) within a wavenumber of 400–4000 cm^−1^.

**Figure 2:**
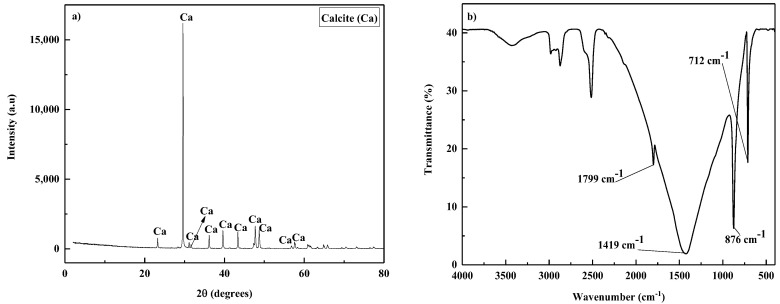
Characterization of a carbonate rock sample by (**a**) XRD and (**b**) FTIR.

The XRF results presented in Table 3 confirm that the carbonate sample’s predominant oxide is calcium oxide (96.7%), and 69.1% of the elemental composition is calcium. The carbonate sample is predominantly calcite, agreeing with the XRD results shown in Figure 2a. Figure 2b shows the FTIR spectrum for the carbonate sample, and various peaks corresponding to the vibration of the carbonate group could be observed. The in-plane and out-of-plane bending vibrations of the CO_3_^2−^ group are shown by peaks at 712 cm^−1^ and 876 cm^−1^, respectively. The asymmetric stretching of the CO_3_^2−^ group is also shown by a peak at 1419 cm^−1^. Then, an absorption peak at 1799 cm^−1^ corresponds to the symmetric stretching and in-plane bending vibration of the CO_3_^2−^ group.

### 2.2. Methods

**Figure 3:**
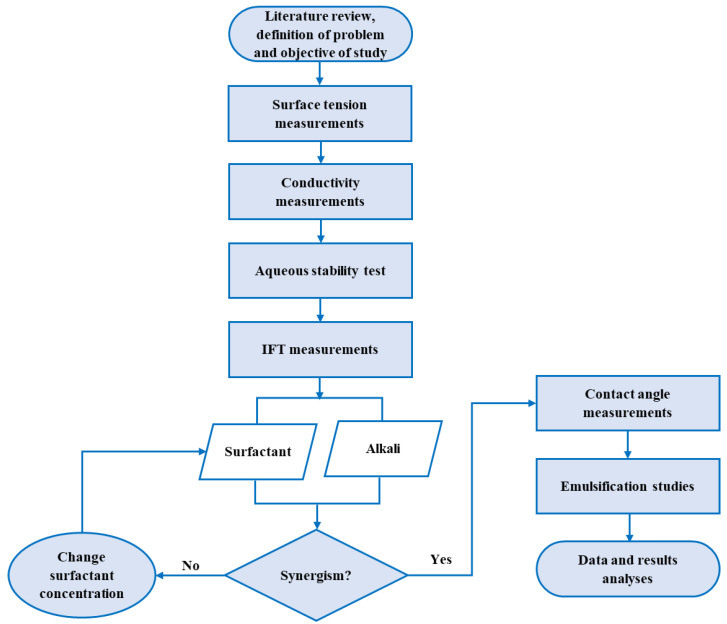
Flow chart of the experimental methods.

The methods deployed in this study include surface tension and conductivity measurements to study the aggregation behaviors and surface activities of the surfactants. The aqueous stability test (i.e., compatibility with brine) was conducted to evaluate the tolerance of the various chemical agents and their combinations to hardness. The interfacial properties and wettability alteration of the surfactants, alkalis and their combination were investigated through IFT and contact angle measurements, respectively. Then, the final step was an emulsification test to corroborate the interfacial properties. Figure 3 shows a flow chart of the experimental methods used in this study.

#### 2.2.1. Surface Tension Measurements

The surface tension measurements of the aqueous solutions of the surfactants were made at different concentrations. The measurements were made using a Rame-Hart Model 260 goniometer (Ramé-hart instrument co., Succasunna, NJ, USA) at room temperature using the pendant drop method. The DROPimage Advance software was used in profile fitting the solution drop suspended from a needle in the air. Single measurements were made repeatedly with a standard deviation of 0.01–0.09 mN/m. Before the measurements, the equipment was calibrated with deionized water, and a value of 74.37 mN/m was found at room temperature.

#### 2.2.2. Conductivity Measurements

Measurements of the electrical conductivities of the surfactant solutions were made at different concentrations with the aid of a Eutech Con 450 conductometer (Poly Scientific, Shah Alam, Malaysia) at room temperature. The surfactants’ concentrations were varied by diluting stock solutions of the surfactants with ethanolamine solution. The solutions were stirred for about a minute and allowed to settle after every dilution before the conductivity measurements. The conductivities were recorded after allowing the reading to stabilize. Conductivity measurements at every concentration were repeated until the values were consistent. The estimated uncertainty was ±0.5 µS/m. Further analyses were made using the mean of three consistent values for each measurement.

#### 2.2.3. Compatibility Test

The compatibility of the 1 wt% alkalis, 0.04 wt% surfactants and their combinations (1 wt% alkali and 0.04 wt% surfactant) with brine was tested. The focus was on evaluating the chemical agents’ hardness tolerance and eliminating scale formation and surfactant precipitation during the flooding experiments. As observed from Table 2, the brine contained an appreciable number of divalent cations. The test was conducted for both the alternative and conventional chemical agents and their combination for comparative purposes. This test mixed aqueous solutions of alkalis, surfactants and AS combinations with the brine. The chemical formulation–brine mixture of a 50:50 volume ratio was used to simulate the contact of the injection and formation water within the reservoir. The mixtures were kept in glass tubes closed tightly, turned up and down a few times to ensure adequate mixing, then left for observation for a week at 80 °C and atmospheric pressure. The evaluation was solely visual, and any sign of precipitation indicated incompatibility.

#### 2.2.4. Interfacial Tension Measurement

The IFT between crude oil and the various aqueous solution of the surfactants was measured using the spinning drop tensiometer (SVT 20, Data physics, Filderstadt, Germany) at room temperature. The measurement process involved the injection of the aqueous phase into a fast exchange capillary tube. The capillary tube was first set to rotate at a very low rotational speed (100–300 rpm); then, the crude oil droplet was injected. The low rotation during the crude oil droplet injection prevented the oil droplet from sticking to the walls of the capillary tube. The tube was then set to rotate at 5000 rpm, which caused the oil droplet to stretch. The elongated oil droplet was profile-fitted using SVT 20 software. The dynamic IFT was recorded at 20-s intervals until equilibrium was reached. The interfacial property in this study was based on the equilibrium IFT. To avoid interference from the former solution, the fast exchange capillary tube was cleaned with toluene, followed by acetone and deionized water, to remove the crude oil and surfactant residues. At ambient conditions, the IFT between crude oil and deionized water was 5.82 mN/m.

#### 2.2.5. Contact Angle Measurements

Wettability alteration studies were done by measuring the contact angle of the surfactant aqueous solution on an oil-aged rock surface. The sessile drop method was applied in measuring the contact angles using the Rame-Hart Model 260 goniometer at ambient conditions. The rock slices described under the Materials section were utilized for the contact angle measurements. Toluene and methanol were used to first clean the slices, then dried. The oil wetness of the slices was induced by aging the slices in crude oil over a fortnight at 80 °C. Afterwards, n-heptane was used to rinse the oil-aged slices, then dried. The slices’ initial wetting conditions were determined from the contact angle of the deionized water. The measurement process involved dropping the surfactant aqueous solution via a needle onto the slice. The solution then formed a sessile drop on the slice, which was analyzed by Young–Laplace fitting. The measurement was done for 10 min. The impact of cross-contamination from traces of the previous solution was mitigated by conducting each measurement on an unaffected part of the rock slice.

#### 2.2.6. Emulsification Test

Emulsification is mostly the prevalent mechanism in surfactant oil recovery processes. Therefore, the emulsifying power of the surfactants–alkali combination and emulsion stability were also studied. The emulsification test involved homogenizing 3 wt% NaCl brine and crude oil using the surfactants at different concentrations as the emulsifying agent. The aqueous solution and crude oil were mixed at a 1:1 ratio in a 25-mL test tube. Homogenization was achieved using T18 digital ULTRA-TURRAX. The homogenized systems were left to equilibrate and observed over time while they disintegrated into their original component at 80 °C. The period of observation was one month, and the percentage reduction in the emulsion phase volume was used to analyze the stability of the emulsions formed. The percentage reduction in the emulsion phase volume is given by:

(1) $$R_{v}=\frac{V_{i}-V_{f}}{V_{i}}\times 100$$

where *V_i_* is the original emulsion phase volume, and *V_f_* is the emulsion phase volume after the period of settling.

## 3. Results and Discussion

### 3.1. Surface Activity and Aggregation Behavior of Surfactants

**Figure 4:**
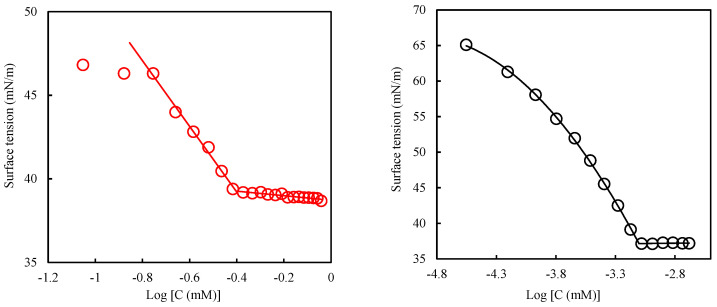
Surface tension versus logarithm of concentration for C_16_mimBr (**left**) and CTAB (**right**) at 25 °C.

The surface activity of the surfactants was studied using the surface tension data. First, the critical micelle concentrations (CMC) of the surfactants were determined. A plot of the surface tension (γ) variation against the log of surfactant concentration (log C) is shown in Figure 4. The observed trend illustrates continuous surfactant adsorption onto the interface between air and water; after which, surface saturation occurs, then self-aggregation [38]. The breaking point on this semi-log plot corresponds to the CMC of the surfactant. The CMCs for the surfactants are given in Table 4. The surface tension method of determining CMC is very versatile, since data about the adsorbed layer at the air–water interface could also be derived [39]. The information on the adsorbed layer at the air–water interface is also presented in Table 4. It is apparent in Table 4 that C_16_mimBr has a lower CMC than CTAB, despite the two having the same hydrophobic tail length. The difference in their CMC is therefore attributed to their headgroup. The planar imidazolium of C_16_mimBr will ensure easier packing into the micelle than the tetrahedral trimethylammonium group of CTAB [40]. Additionally, Wintgens et al. [41] characterized the charge density on the cationic group of the surfactant using the headgroup charge per van der Waals volume. Trimethylammonium has a higher headgroup charge per volume (6.48) than 1-methylimidazolium (5.61). The higher headgroup charge per volume yields increased electrostatic repulsion among the headgroups and, hence, hinders the association of the micelles [41].

Nevertheless, the difference in their CMC is not that significant; hence, a comparable dosage could be used in comparing their performances. CMC determination is routinely deployed to determine the optimum quantity of the surfactants in formulations. In the optimization of oil recovery, surfactant concentrations higher than the CMC are rather used to account for surfactant loss through adsorption on the rock surface.

The surface activity of the surfactants is discussed based on the efficiency and effectiveness in reducing the surface tension. The efficiency refers to the bulk phase concentration required to yield some amount of surface tension reduction. The effectiveness, however, is the maximum surface tension reduction that could be attained regardless of the bulk phase concentration [42]. The efficiency is evaluated using the pC_20_ calculated as:

(2) $${\mathrm{pC}}_{20}=-\log C_{20}$$

where the C_20_ denotes the concentration of surfactant in the bulk phase needed to reduce a pure solvent’s surface tension by 20 mN/m. In other words, the efficiency factor (pC_20_) is the ability of a surfactant to yield a surface pressure of 52 mN/m at the lowest concentration possible [43]. It also depicts the adsorption efficiency [44]. The pC_20_ values from Table 4 show that C_16_mimBr exhibited superior surface tension reduction efficiency compared to CTAB. Since the surface tension reduction efficiency is related to the bulk phase, the observed performance could be explained by the same phenomena that influenced the micellization.

The surface pressure at the CMC denoted by ∏_CMC_ depicts the effectiveness of the surface tension reduction. The surface pressure is the difference in surface tension between a pure solvent and a surfactant solution at a particular concentration. The surface pressure at CMC is therefore shown by Equation (3) below. This parameter is used to measure the surface tension reduction effectiveness, because no profound reduction of surface tension after the CMC is attained.

(3) $$\Pi_{\mathrm{CMC}}=\gamma_{0}-\gamma_{\mathrm{CMC}}$$

The surface pressure values from Table 4 show that CTAB (Π_cmc_ = 33.4 mN/m) is more effective than C_16_mimBr (Π_cmc_ = 34.94 mN/m), though the difference in their surface pressure values is marginal. The effectiveness of the surface tension reduction is dependent on the efficiency and effectiveness of the surfactant adsorption onto interfaces, as shown in Equation (4) [40]:

(4) ΠCMC≈20+2.303nRTΓmlog(CMCC20)

In this equation, n represents the number of solute species with interfacial concentrations that vary with the bulk phase concentration variations, R is the universal gas constant (8.314 JK^−1^mol^−1^) and T is the absolute temperature in kelvin. The CMC/C_20_ ratio, which incorporates the efficiency of adsorption, depicts the spontaneity of micellization relative to adsorption [45]. The CMC/C_20_ ratio value increases because of the structural effect or microenvironmental factor that delays micellization or facilitates adsorption. Therefore, a decrease means the adsorption is hindered or micellization is facilitated [44]. The adsorption effectiveness of the surfactant is depicted by the surface excess concentration (Γ_m_) and the minimum surface area per molecule at the interface at surface saturation (a^s^_m_). These parameters could be calculated with the Gibbs adsorption isotherm [40].

(5) $$\Gamma_{m}=-\frac{1}{2.303\mathrm{nRT}}\left(\frac{d\gamma }{d\log C}\right)$$

(6) $$a_{m}^{s}=\frac{1}{{N\Gamma }_{m}}$$

The value of n is 2 for a dilute solution of 1:1 ionic surfactant [40]. N is Avogadro’s number. From Equation (6), a parallel variation of the CMC/C_20_ ratio and Γ_m_ gives an easier explanation of the observed variation in ∏_CMC_. Since C_16_mimBr has a lower CMC/C_20_ ratio and Γ_m_ than CTAB, micellization is facilitated more in C_16_mimBr, which leads to lesser surfactant molecules available at the interface to ensure more effective surfactant adsorption. This observation implies that a lower concentration of C_16_mimBr is required to achieve the most effective surface tension reduction. A higher concentration, however, is required for CTAB, but it can achieve a more effective surface tension reduction than C_16_mimBr.

### 3.2. Conductivity and Thermodynamic Properties of Aggregation of Surfactants

**Figure 5:**
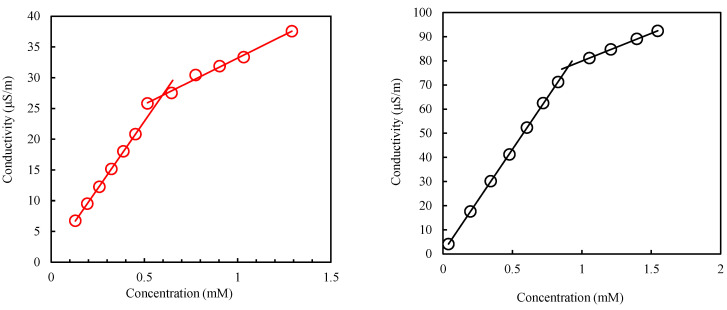
Conductivity versus concentration for C_16_mimBr (**left**) and CTAB (**right**) at 25 °C.

The thermodynamic properties of aggregation for the surfactants were also studied using the conductivity data. The CMC is first determined from the conductivity versus surfactant concentration plot, as shown in Figure 5. Based on Williams’ method, the breaking point on the conductivity variation with the surfactant concentration points to the CMC [46]. The CMC values from the conductivity method are also presented in Table 5. The CMC values showed similar variations as observed in the surface tension method, hence corroborating the CMCs determined. Nevertheless, for each surfactant, the CMC value determined by the conductivity method varies slightly from the surface tension. The methodical differences in CMC determination were explained by Mukerjee and Mysels [47]. The CMC, unlike a property-like a melting point, does not have a sharply defined point above which some properties are qualitatively different from below it. The methodical differences would have been nonexistent or minimal. However, all properties of a solution in the CMC region vary continuously and so do all their derivatives. There is, therefore, a relatively narrow region of concentration in which these changes are most marked. The CMC is therefore a narrow range of concentrations but not a single value.

From the CMC, the Gibbs free energy (ΔG^O^_mic_) of the micellization is computed using Equation (7) [48]:

(7) $$\Delta {G^{O}}_{\mathrm{mic}}=\left(1+\beta \right)\mathrm{RT}\ln X_{\mathrm{CMC}}$$

R and T have their usual meaning in this equation. X_CMC_ represents the CMC in a mole fraction, and β depicts the degree of counterion binding of the micelle. β is derived from the degree of ionization (α). The degree of ionization is the ratio of the slope before CMC to the slope after CMC on the conductivity versus concentration plot. The relationship between the two parameters is given as β = 1 − α [49]. The process of surfactant adsorption onto the interface could also be evaluated through the standard Gibbs free energy of adsorption (ΔG^O^_ads_). It is computed from ΔG^O^_mic_ through Equation (8) [50]:

(8) $$\Delta {G^{O}}_{\mathrm{ads}}=\Delta {G^{O}}_{\mathrm{mic}}-\frac{\Pi_{\mathrm{CMC}}}{\Gamma_{m}}$$

As presented in Table 5, both ΔG^O^_mic_ and ΔG^O^_ads_ are negative, depicting that both the micellization and adsorption processes are spontaneous. The higher values of ΔG^O^_ads_ also show that adsorption is more favored than micellization for both surfactants. In comparison to CTAB (ΔG^O^_mic_ = −47.04 KJmol^−1^ and ΔG^O^_ads_ = −59.62 KJmol^−1^), the lower values of ΔG^O^_mic_ for C_16_mimBr showed that micellization is more spontaneous for CTAB, as the degree of binding is higher, owing to a smaller surface area per headgroup (a^s^_m_, as shown in Table 4). Nevertheless, the higher values of ΔG^O^_ads_ for C_16_mimBr show that its adsorption is more spontaneous.

### 3.3. Compatibility with Brine

A significant limitation in ASP application is scale formation by alkalis and surfactant precipitation due to the divalent cations’ presence (mainly Mg^2+^ and Ca^2+^). The effectiveness and efficiency of most conventional surfactants dwindle in the presence of divalent cations [9]. The precipitates formed reduce the production efficiency through pore blockage [14]. Insoluble scale formation is due to conventionally deployed inorganic alkalis with divalent cations and the reservoir. The insoluble scale formation leads to formation damage, production capacity reduction, lifting system damage and reduction in the average pump-checking period [51,52,53]. Therefore, it is vital to consider the hardness tolerance of surfactants in their EOR applications [54]. The rule of thumb is to maintain the concentrations of divalent ions below 10 ppm for efficient alkali application [11]. Therefore, massive pre-flush and other costly measures, such as water treatment by ion exchange or other techniques for softening brine, are required to ensure efficient oil recovery [11,55]. The hardness tolerance of ionic surfactants is also improved by adding nonionic surfactant or alcohol to their formulations [54].

**Figure 6:**
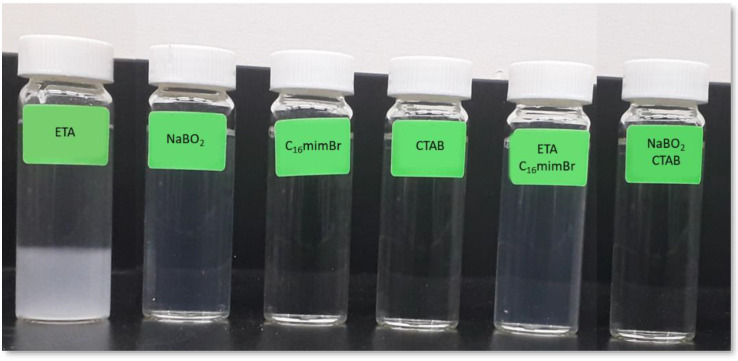
Compatibility of the chemical solutions with brine.

The compatibility of alkalis, surfactants and AS formulations with brine is shown in Figure 6. The ETA was incompatible with brine, while NaBO_2_ formed a stable and clear solution with brine. With higher divalent cation concentrations, the hardness tolerance of ETA is exceeded. The ethanolamines, diethanolamine and triethanolamine are more tolerant to hardness than ETA [56]; nevertheless, ETA has been proven to have a better synergistic effect with the surfactant in IFT reduction [12]. Therefore, ETA was the choice for this study among the ethanolamines. NaBO_2_, on the other hand, is known to sequester divalent ions [57,58]. C_16_mimBr and CTAB were not expected to form a precipitate with brine, since they are cationic surfactants [14]. Both the combination of ETA with C_16_mimBr and NaBO_2_ with CTAB formed a clear solution with brine. Therefore, no precipitations and formation damage are expected in their flooding process.

### 3.4. Interfacial Properties

**Figure 7:**
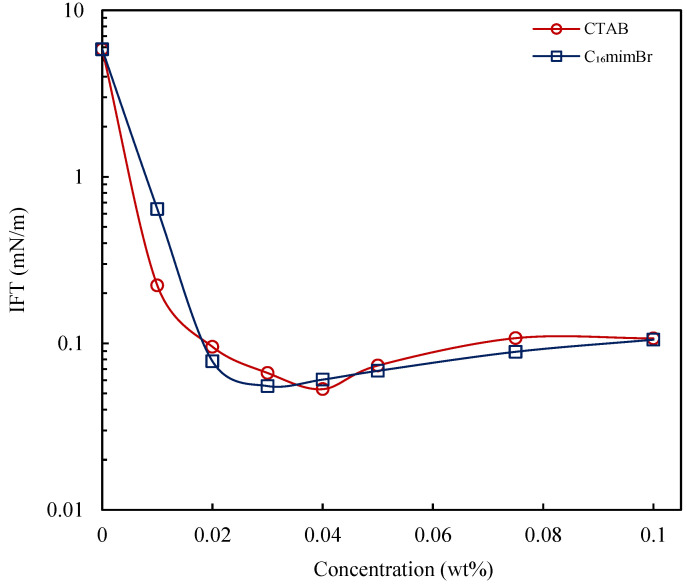
Aqueous–crude oil IFT variation with the surfactant concentration at 25 °C.

IFT reduction is known to be a predominant mechanism in the application of the surfactant and alkali for enhancing oil recovery. The interfacial properties of these chemical agents are therefore vital in developing an optimum formulation for improving oil recovery. Herein, the performance of the ETA–C_16_mimBr combination in reducing IFT is evaluated in comparison to a NaBO_2_–CTAB combination, both in a deionized water and brine solution. The effect of temperature is evaluated as well. The IFT reduction capability of C_16_mimBr is first compared to CTAB, as presented in Figure 7. C_16_mimBr had a similar IFT reduction capability as CTAB, with a minimum IFT (IFT_min_) of 0.055 mN/m (at 0.03 wt% C_16_mimBr concentration), and CTAB had an IFT_min_ of 0.053 mN/m (at 0.04 wt% CTAB concentration). This observation confirms their surface activity, as explained in Section 3.1. C_16_mimBr attains IFT_min_ at a lower concentration, owing to the facilitated micellization process. Due to the delayed micellization in CTAB, there more surfactant molecules available at the oil–water interface to reduce IFT further. The predominant surfactant feature that enhances the IFT reduction capability is the alkyl chain length [40]. The two cationic surfactants have the same alkyl chain length, which masks the difference in the interfacial properties caused by the differences in their headgroups, hence the similarities in their interfacial properties.

#### 3.4.1. Effect of Alkali

**Figure 8:**
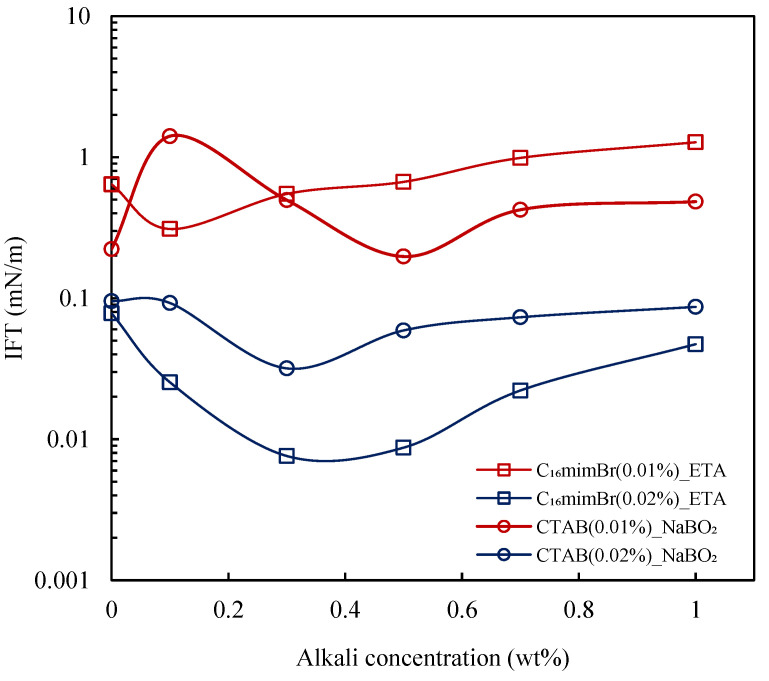
Effect of alkali on the oil–aqueous IFT.

The combination of alkali and surfactant is known to yield synergistic performances [3,34,59,60]. Based on the IFT reduction studies of the surfactants, concentrations of 0.02 wt% and 0.01 wt% were chosen. The two cationic surfactants achieved IFT_min_ at different concentrations; therefore, 0.02 wt% is chosen as a common concentration to investigate the effect of alkalis. The IFT reduction of alkalis at various surfactant concentrations is presented in Figure 8. At a 0.02 wt% surfactant concentration, a synergistic effect was observed for both surfactants. C_16_mimBr at 0.02 wt% reached an IFT_min_ of 7.6 × 10^−3^ mN/m at a 0.3 wt% ETA concentration. Comparing this value to the IFT_min_ attained by C_16_mimBr without alkali (i.e., IFT_min_ of 0.055 mN/m), there is evidence of synergism in the combination of C_16_mimBr and ETA. An ultra-low IFT is achieved at a lower surfactant concentration upon the addition of alkali. The combination of NaBO_2_ and CTAB also yielded an IFT_min_ of 0.0318 mN/m (at a lower surfactant concentration), which is better than the IFT_min_ attained by CTAB without alkali. This observation agrees with the report of Kumar and Mandal [61] on the IFT reduction capability of the CTAB–NaBO_2_ combination. The subsequent reduction in surfactant concentration to 0.01 wt% also yielded a synergistic effect, as shown in Figure 8. Nevertheless, the IFT reduction at a 0.02 wt% surfactant concentration was better.

#### 3.4.2. Comparison of Synergism

**Figure 9:**
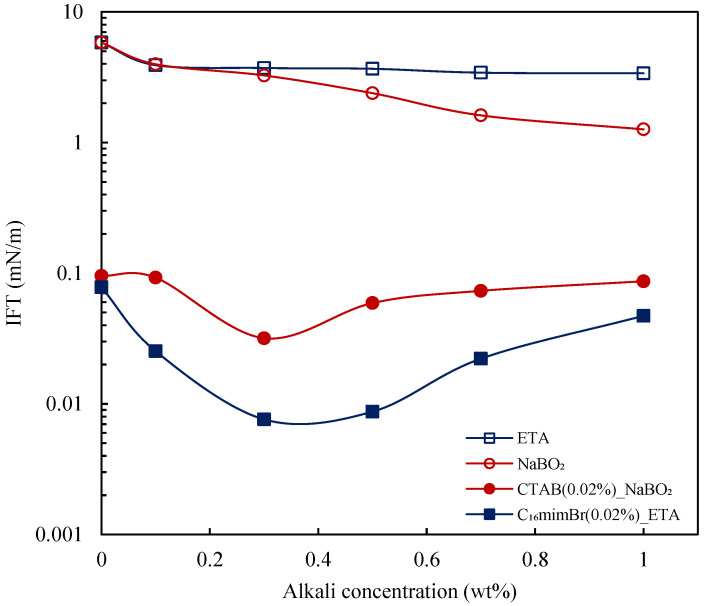
Aqueous–crude oil IFT variation with alkali concentration for chemical solutions at 25 °C.

The IFT reduction capability of the ETA–C_16_mimBr combination is compared to the NaBO_2_–CTAB combination to explore its performance. Firstly, the alkali performance in IFT reduction is discussed and presented in Figure 9. It is well-established in the literature that inorganic alkalis (NaBO_2_) reduce IFT via the formation of an in situ surfactant through the deprotonation of acids. This phenomenon is caused by the ability of the inorganic alkalis to form carbonic acid that removes free H^+^ ions from the solution [3]. In this study, the ETA did not reduce the IFT as much as reported in the literature [16]. As explained by Bai et al. [16], ETA renders the aqueous solution basic through the amine group in its structure, which generates an in situ surfactant by reacting with the saponifiable component of crude oil. They also explained that ETA has an amphiphilic structure owing to its alkyl and hydroxyl group and, hence, could act as a surfactant. Both alkalis yielded a low IFT reduction performance due to the crude oil’s low acid content (TAN = 0.01 mg KOH/g) though the NaBO_2_ performed better [62]. This observation proves that the amphiphilic nature of ETA does not guarantee the ability to reduce IFT. Therefore, ETA could not be considered as a surfactant capable of reducing IFT on its own. A longer alkyl chain length is a prerequisite for effective IFT reduction by an amphiphilic substance [40].

Nevertheless, the ETA–C_16_mimBr system reduced IFT better than the NaBO_2_–CTAB system showing a better synergism, as seen in Figure 9. The performance could be attributed to in situ soap generation. Inorganic alkalis (NaBO_2_) generate cationic petroleum carboxylate, but the nonionic alkanolamide soap generated by organic alkalis (ETA) gives a better synergy with the surfactants [22,63]. Furthermore, ETA and the generated in situ soap form a mixed surfactant system with the surfactant, which ensures tighter and better interfacial packing leading to the improved effectiveness of IFT reduction [12,16]. In the NaBO_2_–CTAB system, the low acidic crude oil component limits the in situ soap generation. Therefore, there would be insufficient saponin to form the mixed surfactant system with the CTAB and, hence, less synergistic performance.

#### 3.4.3. Effect of Salinity

**Figure 10:**
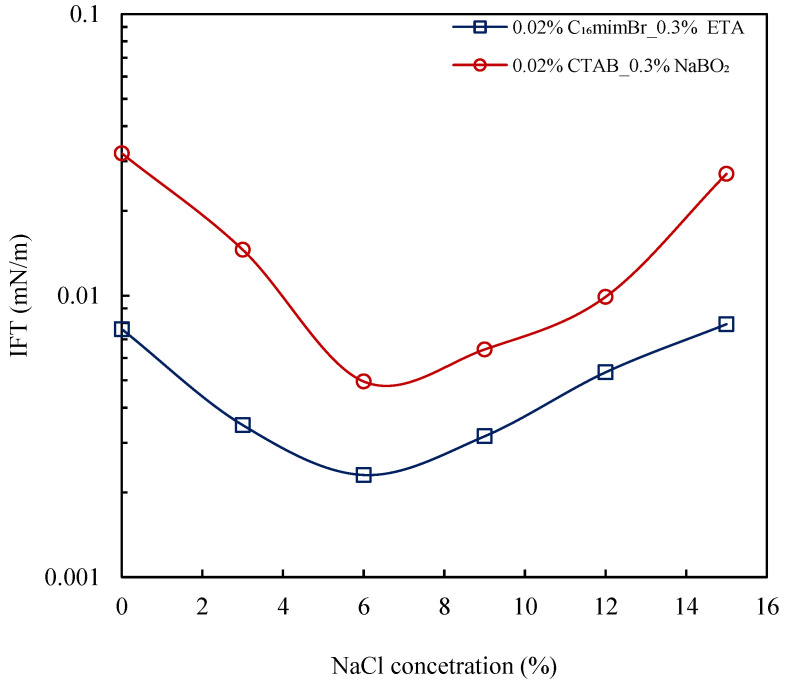
Salinity effect on IFT reduction by AS formulations at 25 °C.

The salinity has a significant impact on the interfacial properties. Generally, salt yields a synergistic effect with surfactants in reducing the IFT [22,23]. The effect of salt concentrations on IFT reduction by both AS formulations is shown in Figure 10. Both formulations improved in their IFT reduction capabilities in the presence of salt. The improvement in IFT reduction is attributed to a reduction in electrical repulsion due to the presence of opposite ions of the salt. The salt ions may also present competition with cations and anions of the surfactants in attracting water molecules, therefore reducing the solubility of surfactants [64]. The increase in salt concentration first resulted in further reduction in IFT to ultra-low levels at an optimum salinity of 6 wt% NaCl. The ETA–C_16_mimBr system attained an IFT_min_ of 2.3 × 10^−3^ mN/m, while the NaBO_2_–CTAB system attained an IFT_min_ of 4.95 × 10^−3^ mN/m. Further, an increase in salt concentration beyond the optimal salinity resulted in increasing the IFT. This observation could be attributed to the desorption of surfactant molecules at a high salinity and their subsequent dissolution into the oil phase [65]. Nevertheless, the IFT of the ETA–C_16_mimBr system remained ultra-low even at very high salinity (i.e., 15 wt% NaCl concentration). This observation shows that the ETA–C_16_mimBr system would be a good candidate for application in high salinity conditions.

#### 3.4.4. Effect of Temperature

**Figure 11:**
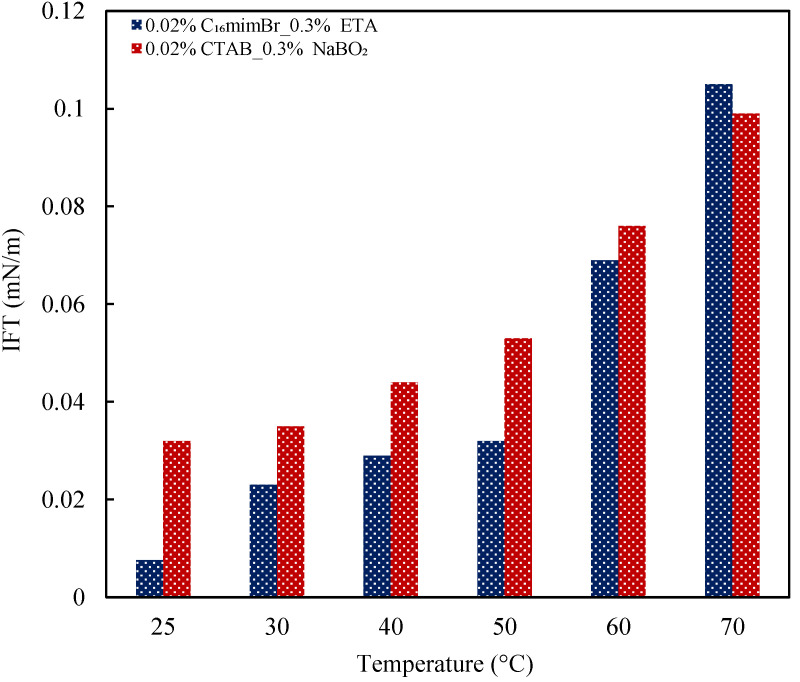
Effect of temperature on IFT reduction by AS formulations.

The IFT reduction capabilities of the surfactants are best explained based on their effectiveness and efficiency of adsorption onto the oil–water interface. Generally, for ionic surfactants, a temperature increase causes a decrease in adsorption effectiveness and efficiency. This observation could be ascribed to the improved solubility of surfactant molecules at elevated temperatures, limiting the concentration of surfactant molecules at the oil–water interface [40]. Nevertheless, the literature has reported contradicting findings on the IFT response to temperature [66]. From the observation made in this study during the IFT measurement process confirmed by the study of Okasha [67], the observed temperature effect on IFT is predominantly due to the type of crude oil. IFT between a dead oil and brine system reduces with the temperature increase, while IFT between a live oil and brine system increases [67]. As observed in this study, with increasing the temperature, dissolved gas in crude oil expands, resulting in an increase in density difference and the radius of the crude oil droplet. Referring to the relation for determining the IFT, as shown in Equation (9) [23], the IFT is expected to increase. The IFT variations with the temperatures for both AS formulations are shown in Figure 11. Both formulations exhibited increased IFT with the temperature increase. The live oil effect overshadowed the performance of the formulations at high temperatures. Therefore, the IFT reduction performance of both formulations at elevated temperatures is further investigated through emulsification studies, as low IFT is required to generate stable emulsions.

(9) $$\sigma =\frac{\omega^{2}R^{3}\Delta \rho }{4}$$

where ω is the angular velocity, R is the crude oil droplet radius and Δρ is the difference in density between an aqueous solution of surfactant and crude oil.

### 3.5. Wettability Alteration Characteristics

A favorable displacement is achieved in the multiphase flow of oil and water in the reservoir when the displacing fluid preferentially wets the rock surface. However, not all reservoirs are wet with water. Due to the prolonged oil storage within reservoirs, most oil reservoirs are either intermediately wet or wet with oil [68,69]. The wetting process involves surfaces and interfaces. Therefore, the ability to modify the wetting power of water or an aqueous solution is a surface property exhibited by all surfactants, yet to a greatly varied extent [40]. Surfactant and/or alkali application in EOR also yields favorable oil displacement by ensuring the aqueous phase preferentially wets the rock surface.

#### 3.5.1. Wettability Alteration by Surfactants

**Figure 12:**
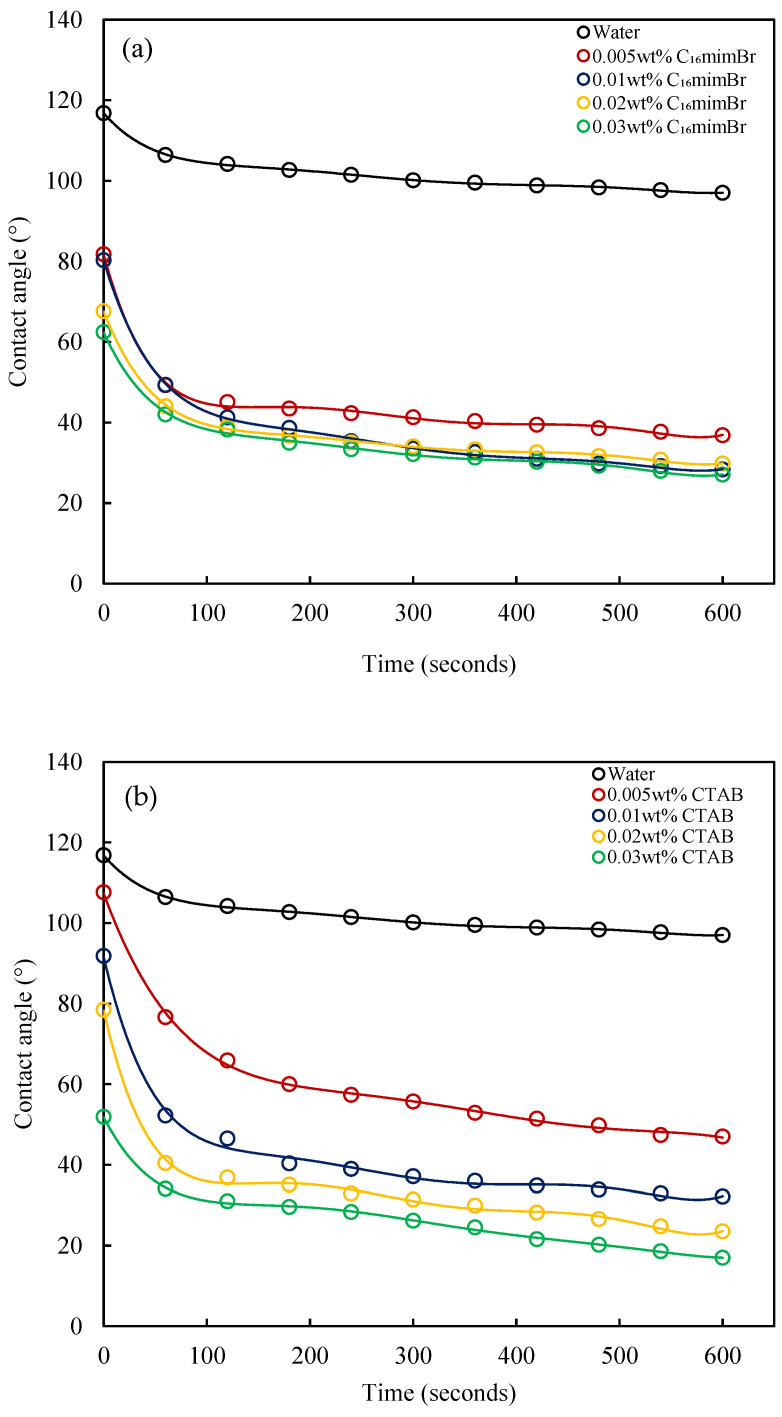
The dynamic contact angle of (**a**) C_16_mimBr and (**b**) CTAB on a carbonate surface.

The dynamic contact angle at various concentrations for C_16_mimBr and CTAB is shown in Figure 12. From both figures, it is observed that the contact angle of water varies from 116° to 97° in 10 min. This means the carbonate surface is wet with oil. From Figure 12a, it is observed that the contact angle decreases significantly with the increasing C_16_mimBr concentration. Beyond a 0.01 wt% concentration, the decrease in the contact angle becomes marginal. However, for the CTAB solutions (Figure 12b), the contact angle reduced further with the increasing CTAB concentration. Comparing the initial and final contact angles at various concentrations for C_16_mimBr and CTAB, it is apparent that the CTAB solutions exhibited better wettability alteration capabilities than C_16_mimBr. The surface activity study showed that CTAB has a higher Γ_m_, which means more surfactant molecules are available at the solid–liquid interface to alter the rock surface wettability. Both surfactants have positive headgroups, and with the positive charge surface of carbonate, the observed wettability alteration is mainly attributed to the ion pair mechanism. The negative components of crude oil, predominantly fatty acids and carboxylate anions, adsorb onto the positive surface of carbonate and render it wet with oil [70]. The positive headgroups of C_16_mimBr and CTAB form ion pairs with the negative crude oil component adsorbed on the carbonate surface and detach them, leaving the rock surface wet with water [33].

#### 3.5.2. Wettability Alteration by Alkalis

The mechanisms in the application of alkali to enhance oil recovery are displacement through low IFT, breaking of a rigid film and wettability reversal [71]. However, wettability reversal becomes the preponderant mechanism in reservoirs with light crude oil [72]. In alkali flooding, the properties of the crude oil determine the predominant mechanism. The mechanisms, therefore, are associated with the general classes of compounds, like asphaltenes, acids, etc., in the crude oil [72]. This study used a crude oil with a low acidic content; hence, wettability reversal by an alkali would be an essential mechanism in recovering this crude oil type. Nevertheless, alkali application in carbonate reservoirs is limited due to the presence of anhydrite and gypsum, which cause precipitation problems. Carbonate reservoirs also contain brine with higher divalent cation concentrations [3].

**Figure 13:**
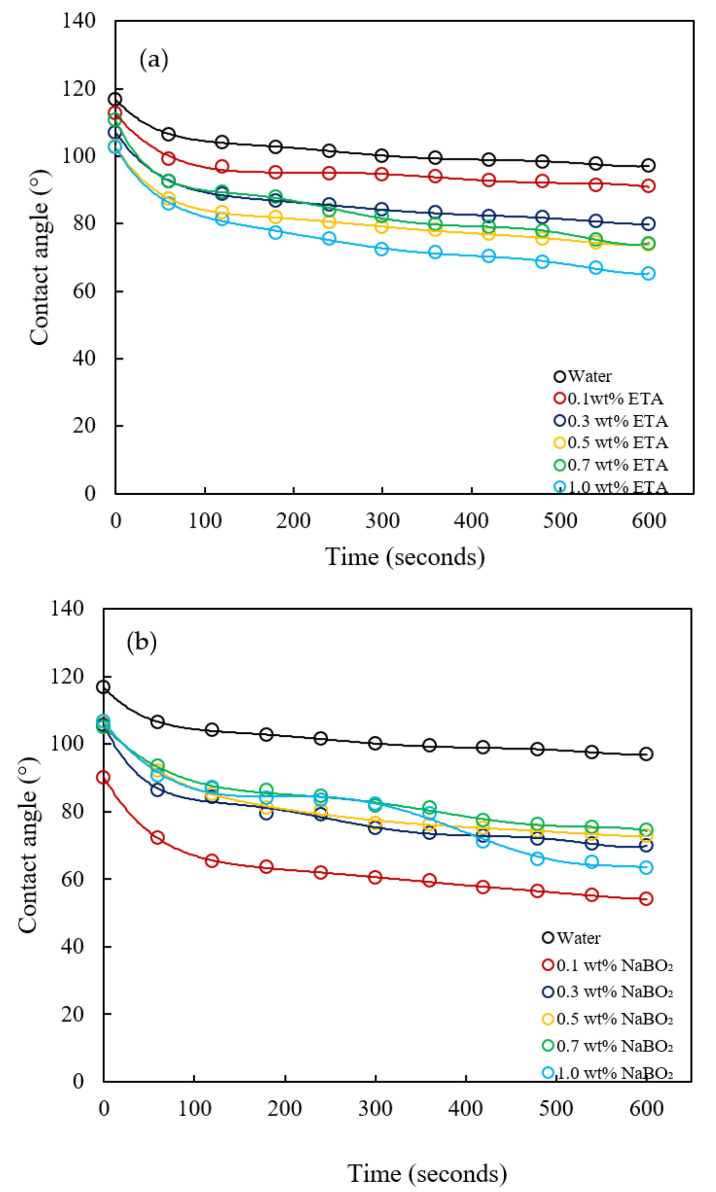
The dynamic contact angle of (**a**) ETA and (**b**) NaBO_2_ on a carbonate surface.

Therefore, the wettability reversal by inorganic alkalis like NaOH and Na_2_CO_3_ in carbonate reservoirs are limited in the literature. Among the alternative alkalis to alleviate the precipitation problems are NaBO_2_ and organic alkalis [9]. Nevertheless, their wettability alteration capabilities in carbonate formations are not reported in the literature. Herein, the wettability reversal by NaBO_2_ and ETA is explored through contact angle measurements. The dynamic contact angles at different concentrations of ETA and NaBO_2_ are presented in Figure 13. As shown above, the contact angle variations with time for water are from 116° to 97°, indicative of a wet oil condition. From Figure 13a, it could be observed that, except for the anomaly at a 0.7 wt% ETA concentration, further reduction in the contact angle is observed with the increasing ETA concentration. At a 1.0 wt% ETA concentration, the dynamic contact angle varied from 102° to 65°, depicting the ETA capability in altering the carbonate surface wettability. On the other hand, NaBO_2_ exhibited the most effective wettability alteration capability at a 0.1 wt% concentration (dynamic contact angle varied from 90° to 54°). A further increase in the concentration resulted in reduced effectiveness in the contact angle reduction.

**Figure 14:**
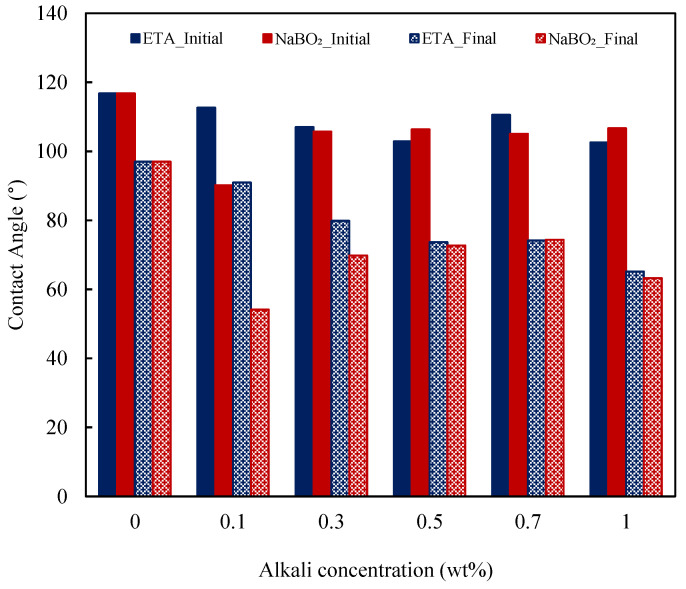
Initial and final contact angle variations with the alkali concentrations for ETA and NaBO_2_.

Comparatively, NaBO_2_ exhibited better wettability alterations than ETA, though the difference in their performances was marginal, as demonstrated in Figure 14. The figure shows a comparison of the initial and final contact angles at different alkali concentrations. Both alkalis render the surface of the carbonate rock moderately water wet. Various wettability reversal mechanisms have been proposed for inorganic alkalis; yet, the well-established ones are ion exchange and alkali interactions with rock [3]. The wettability reversal could also occur through alterations of the oil–water or liquid–solid IFT [72]. ETA, being a weak alkali, would have a weak interaction with the rock; therefore, wettability reversal is not as effective as in the case of inorganic alkalis [73]. This interaction yields hydrogen bonding between its hydroxyl group and the rock minerals, replacing the polar compounds adsorbed on the rock surface [17].

#### 3.5.3. Synergism in Wettability Alteration

**Figure 15:**
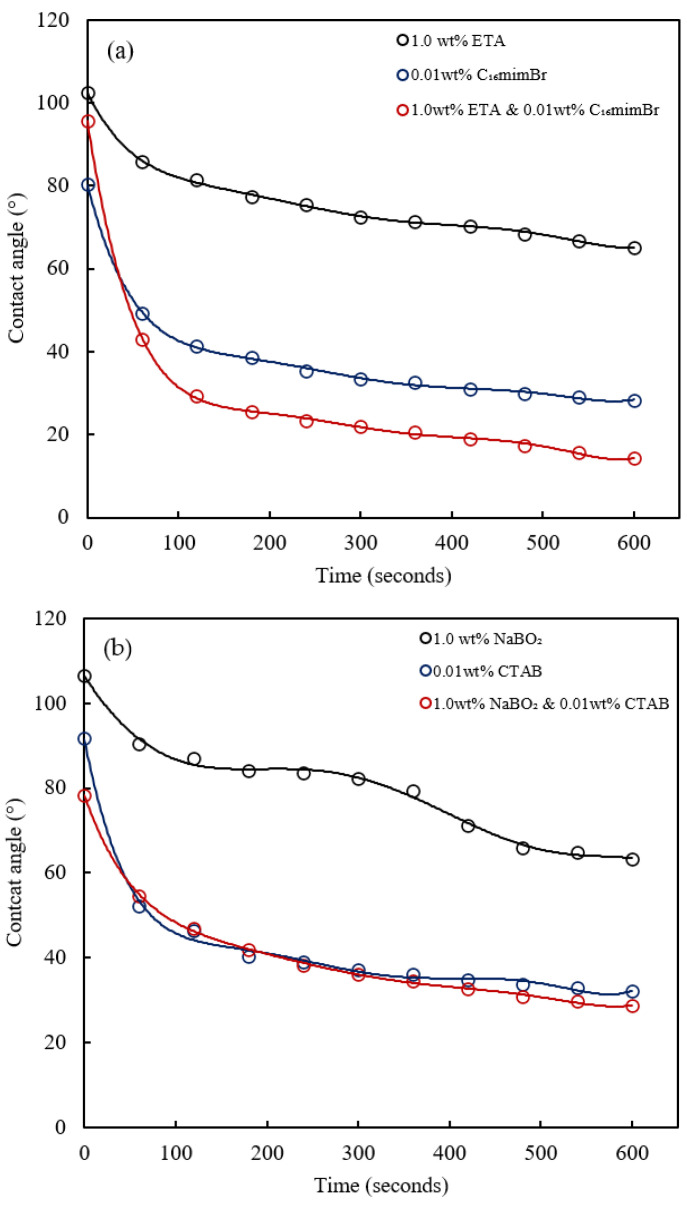
The dynamic contact angle of (**a**) ETA, C_16_mimBr and the ETA–C_16_mimBr system and (**b**) NaBO_2_, CTAB and the NaBO_2_–CTAB system on a carbonate surface.

The addition of alkalis also augments the wettability alteration performances of the surfactants [74]. The synergistic performance of the alkali–surfactant combination in wettability alteration is evident both in the contact angle reduction [61,75] and spontaneous imbibition [76]. The AS systems formulated to explore the synergism in the wettability alteration are composed of 1.0 wt% alkali and 0.01 wt% surfactant. The dynamic contact angle of the two AS formulations compared with their chemical agents is shown in Figure 15. The wettability alteration by the ETA–C_16_mimBr system showed evidence of synergism. On the other hand, the addition of NaBO_2_ to CTAB yielded a marginal improvement in the performance of CTAB. The observed synergism is attributed to the combined effect of different mechanisms of wettability alterations by surfactants and alkalis [73].

Alkalis react with an acidic component of crude oil to generate soap in situ, as explained under interfacial properties. A mixed surfactant system forms between the in situ soap and the surfactant with enhanced wetting power [40]. Nevertheless, the low acidic content of the crude oil will result in the generation of insufficient soap; therefore, this phenomenon is likely to be less effective. This explains the marginal performance between CTAB and the NaBO_2_–CTAB system. On the other hand, ETA’s amphiphilic nature means it could form a mixed surfactant system with C_16_mimBr. The nonionic ETA could increase the C_16_mimBr mobility, resulting in rapid molecular diffusion to the wetting front [77]. This phenomenon improves the wetting power of C_16_mimBr, hence the observed improved performance in the ETA–C_16_mimBr system. Another explanation could be the improved solubilization of C_16_mimBr by ETA, which enhances its wetting properties [78]. The ETA–C_16_mimBr system, therefore, exhibited better wetting power than the NaBO_2_–CTAB system.

### 3.6. Emulsification Studies

The emulsification mechanism causes oil to be entrained and produced in water. The oil droplets could also merge and block pores to improve the sweep efficiency by the emulsification and entrapment mechanism [3,79]. Surfactants facilitate the dispersion and emulsification of particles and droplets due to their amphiphilic nature. Nevertheless, emulsions generally demonstrate kinetic stability, since there is the tendency for the system to disintegrate and reduce the interfacial area and energy [80]. Emulsification and the stabilization of emulsions require low IFT between the immiscible fluids and the application of an adequate shear to promote homogenization [81].

**Figure 16:**
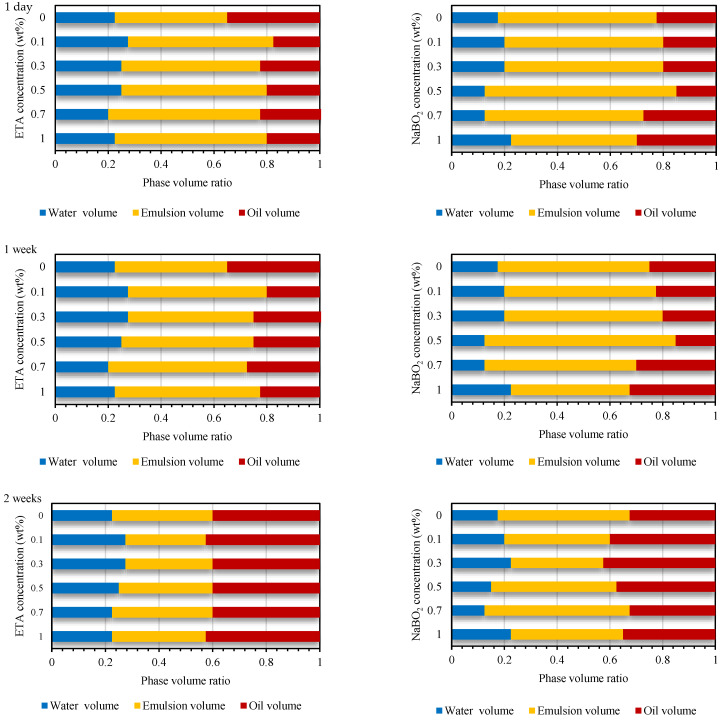

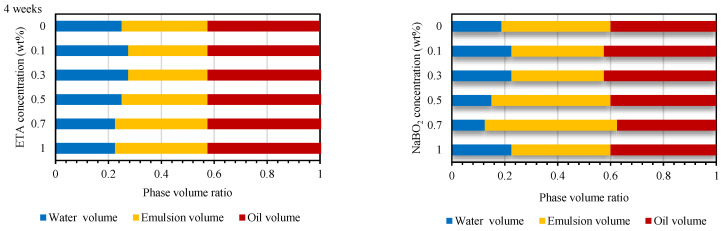
Emulsion stability of the ETA–C_16_mimBr (**left**) and NaBO_2_–CTAB (**right**) systems at various alkali concentrations at 80 °C.

To corroborate the observed IFT reduction capability of the various AS formulations and the effect of salinity and elevated temperature on their interfacial properties, emulsion stability studies are conducted at a 3 wt% NaCl concentration and 80 °C temperature. Aqueous solutions of surfactants at 0.02 wt% and alkali at concentrations 0 to 1 wt% and electrolytes were mixed with the crude oil and homogenized at 5000 rpm for 10 min. The emulsion stability is inferred from the phase volume ratio variations observed for one month, as presented in Figure 16.

With the addition of a surfactant and/or alkali, the emulsification mechanism could be based on the surface tension theory [40]. The reduction of IFT by the surfactants and alkali reduced the amount of mechanical work required to break the inner phase into dispersed particles. Both C_16_mimBr and CTAB are known to form emulsions with smaller droplet sizes and narrow droplet size distributions yielding stable emulsions [37,61]. The percentage reduction in the emulsion phase volume over one month for C_16_mimBr was ~24%, while that of CTAB was ~31%. Nevertheless, the NaBO_2_–CTAB system formed more stable emulsions with a percentage reduction in the emulsion phase volume in the range of ~17–42%, while the ETA–C_16_mimBr system also formed a stable emulsion with an emulsion phase volume reduction in one month of ~39–45%. For both systems, it was observed that emulsion stability decreased upon the addition of alkali, then improved with the increasing alkali concentration.

The emulsions formed are oil in water (o/w)-type emulsions. The emulsion type formed is due to the hydrophile–lipophile balance (HLB) of the surfactants [82]. The disintegration of the emulsion phase also resulted in an increase the oil phase, as seen in Figure 16. This observation means oil is the dispersed phase. With the formation of o/w emulsions, the stability of the emulsions formed is due to the existence of an electrical or steric barrier to coalesce on the dispersed droplets [40]. The source of the charge on the dispersed droplets is the adsorbed layer of the surfactant with its hydrophilic head oriented toward the aqueous phase. Therefore, the charge on the oil droplets that yields the repulsive force to keep them dispersed is that of the amphipathic ion of C_16_mimBr and CTAB.

Another factor that reduces the rate of coalescence of the oil droplets is the mechanical strength of the interfacial film surrounding the oil droplets. The stronger the film, the less chance of coalescence upon the collision of oil droplets. The strength of the interfacial film is dependent on the tighter packing of surfactant molecules on the oil/water interface. The packing is tighter with the increasing alkyl chain length of the surfactants, and that explains why both systems formed stable emulsions. NaBO_2_ as an inorganic alkali would act as a salt and reduce the electrostatic repulsion among the CTAB headgroups, yielding tighter packing and more stable emulsions. For the ETA–C_16_mimBr system, a mixed surfactant system is formed between ETA and C_16_mimBr, which also yields tighter packing and stable emulsions, but the emulsion stability study proved that the former phenomenon is more effective. Therefore, based on the emulsification studies, it could be concluded that both AS formulations are stable at elevated temperatures.

## 4. Conclusions

A combination of C_16_mimBr and ETA was investigated for its possible application in alkali–surfactant flooding. The two chemical agents have been proposed in previous studies as alternatives to conventional surfactants and alkalis, respectively. It is believed that their application could mitigate the effect of the limitations associated with their conventional counterparts. Thus, this proposed AS formulation was studied in comparison to a conventional AS formulation made of CTAB and NaBO_2_. The following conclusions could be deduced from this study:

The study confirmed that C_16_mimBr and CTAB have similar aggregation behaviors and surface activities.

Though ETA exhibited an incompatibility with brine, its combination with C_16_mimBr proved to eliminate the issue of scaling and surfactant precipitation. The conventional chemicals deployed in this study were also compatible with brine, as reported in the literature.

The addition of the alkalis to the surfactants exhibited a synergistic performance in IFT reduction for both AS formulations. The ETA–C_16_mimBr system proved to be better than the NaBO_2_–CTAB system in IFT reduction, yielding an ultra-low IFT of 7.6 × 10^−3^ mN/m. The ETA–C_16_mimBr system also showed synergism in the presence of salt and maintained an ultra-low IFT even at a high salinity of 15 wt% NaCl concentration. The IFT increased with the temperature due to the dissolved gases in crude oil.

The ETA–C_16_mimBr combination also exhibited a synergistic performance in altering the surface of carbonate rock, while the effect of NaBO_2_ on the wettability alteration capability of CTAB was not significant.

The emulsification studies confirmed the synergism in the IFT reduction performance of the AS formulations and showed that the ETA–C_16_mimBr system could form very stable emulsions at high-temperature conditions just like the NaBO_2_–CTAB system. Thus, this study showed that a combination of surface-active ionic liquid and organic alkali have excellent potential in enhancing the oil recovery in carbonate reservoirs at high-salinity, high-temperature conditions in carbonate formations.

## Figures and Tables

**Table 1:** Details of the experimental materials.

Type	Materials	Purity *	Supplier
Surfactants	1-hexadecyl-3-methyl imidazolium bromide	AR, over 99%	Career Henan Chemical Co (Zhengzhou, China)
	Cetyltrimethylammonium bromide	AR, over 99%	Acros Organics (Semenyi, SGR, Malaysia)
Alkalis	Monoethanolamine	~99.5–100%	R and M chemicals (Subang Jaya, Malaysia)
	Sodium metaborate tetrahydrate	AR, 99.5%	Sigma-Aldrich (Petaling Jaya, Malaysia)
Salts	Strontium chloride hexahydrate, SrCl_2_.6H_2_O	AR, 99%	Merck Chemicals (Petaling Jaya, Malaysia)
	Calcium chloride dihydrate, CaCl_2_.2H_2_O	AR, 99.5%	R and M chemicals (Subang Jaya, Malaysia)
	Magnesium chloride hexahydrate, MgCl_2_.6H_2_O	AR, 99.5%	R and M chemicals (Subang Jaya, Malaysia)
	Potassium chloride, KCl	AR, 99.5%	R and M chemicals (Subang Jaya, Malaysia)
	Sodium chloride, NaCl	AR, 99.5%	R and M chemicals (Subang Jaya, Malaysia)
	Sodium bicarbonate, NaHCO_3_	AR, over 99%	R and M chemicals (Subang Jaya, Malaysia)
	Sodium sulfate, Na_2_SO_4_	AR, over 99%	R and M chemicals (Subang Jaya, Malaysia)
Oleic phase	Crude oil	-	Portray (M) SDN BHD (Petaling Jaya, Malaysia)

* AR is analytical reagent.

**Table 2:** Brine and crude oil compositions and properties.

Salt	Concentration (g/L)	Crude Oil Composition	% Weight
NaCl	23.9667	Saturates	55.6
KCl	0.7150	Aromatics	24.6
MgCl_2_·6H_2_O	10.8322	Resins	16.3
CaCl_2_·2H_2_O	1.5737	Asphaltenes	3.5
SrCl_2_·6H_2_O	0.0201		
Na_2_SO_4_	4.0663		
NaHCO_3_	0.2189		
Properties	Brine	Crude oil	
Density (g/mL) @ 25 °C	1.0229	0.8404	
Density (g/mL) @ 80 °C	0.98281	0.809	
Viscosity (mPa.s) @ 25 °C	1.041	13.6	
Viscosity (mPa.s) @ 80 °C	0.5334	6.3	
Salinity (mg/L)	41392.9		
Total acid number (mg KOH/g)		0.01	

**Table 3:** Carbonate rock composition (XRF analysis).

Oxide	Concentration (%)	Elemental Composition	Concentration (%)
CaO	96.7	Ca	69.1
MgO	1.18	Mg	0.710
SiO_2_	0.673	Si	0.315
P_2_O_5_	0.667	P	0.291
Al_2_O_3_	0.258	Fe	0.180
Fe_2_O_3_	0.257	Al	0.137
K_2_O	0.0868	K	0.0720
SO_3_	0.0789	Cl	0.0650
Cl	0.0650	S	0.0316
SrO	0.0299	Sr	0.0253

**Table 4:** Parameters obtained from the surface tension data at 25 °C.

Surfactant	CMC (mM)	ϒ_cmc_ (mN/m)	pC_20_	CMC/C_20_	Π_cmc_ (mN/m)	Γ_m_ (µmol/m^2^)	a^s^_m_ (Å^2^)
C_16_mimBr	0.54	38.6	3.78	3.6	33.4	2.03	81.6
CTAB	0.84	37.01	3.67	3.93	34.94	2.78	59.73

**Table 5:** Thermodynamic parameters derived from conductivity data.

Surfactant	CMC (mM)	α	β	∆G^O^_mic_ (kJ/mol)	∆G^O^_abs_ (kJ/mol)
C_16_mimBr	0.60	0.34	0.66	−46.97	−63.42
CTAB	0.85	0.27	0.73	−47.04	−59.62

## Data Availability

Not applicable.

## Sample Availability

Samples are not available from the authors.
